# Distinct Properties of Long-Term Potentiation in the Dentate Gyrus along the Dorsoventral Axis: Influence of Age and Inhibition

**DOI:** 10.1038/s41598-017-05358-1

**Published:** 2017-07-11

**Authors:** An Schreurs, Victor Sabanov, Detlef Balschun

**Affiliations:** KU Leuven, Faculty of Psychology and Educational Sciences, Brain & Cognition, Laboratory of Biological Psychology, Leuven, Belgium

## Abstract

The hippocampus is important for spatial navigation, episodic memory and affective behaviour. Increasing evidence suggests that these multiple functions are accomplished by different segments along the dorsal-ventral (septal-temporal) axis. Long-term potentiation (LTP), the best-investigated cellular correlate of learning and memory, has distinct properties along this axis in the CA1 region, but so far, little is known about longitudinal differences in dentate gyrus (DG). Therefore, here we examined potential dorsoventral differences in DG-LTP using *in vitro* multi-electrode array recordings. In young mice, we found higher basal synaptic transmission in the dorsal DG, while the LTP magnitude markedly increased towards the ventral pole. Strikingly, these differences were greatly reduced in slices from middle-aged mice. Short-term plasticity, evaluated by paired-pulse ratios, was similar across groups. Recordings in the presence and absence of GABA_A_-receptor blocker picrotoxin suggested a higher inhibitory tone in the ventral DG of young mice, confirmed by an increased frequency of miniature inhibitory postsynaptic currents. Our findings support the view that the hippocampus contains discrete functional domains along its dorsoventral axis and demonstrate that these are subject to age-dependent changes. Since these characteristics are presumably conserved in the human hippocampus, our findings have important clinical implications for hippocampus- and age-related disorders.

## Introduction

It is well established that the hippocampus is involved in cognitive functioning, especially explicit and spatial memory, and emotional behaviours like stress and anxiety. Besides the well-characterized subregions Cornu Ammonis (CA) 1–3 and dentate gyrus (DG), the hippocampus can be further functionally divided along its longitudinal axis in dorsal (septal), intermediate and ventral (temporal) segments. The existence of such segregation is supported by an increasing number of behavioural, anatomical, molecular and gene expression studies (as reviewed in refs 1 and 2). The segments have unique connections to other brain regions, different place cell and place field properties, and lesions in either dorsal or ventral hippocampus lead to distinct functional deficits in behavioural tasks. For instance, the dorsal hippocampus was shown to be crucial for spatial memory^3^, whereas the ventral hippocampus interacts with the hypothalamus and amygdala, making it a regulator of stress and anxiety^4^. DG granule cells were further reported to control separate properties of learning and anxiety, such as encoding and retrieval of contextual fear memories, depending on their position along the longitudinal axis^5^, and their cell density decreases towards the ventral end^6^.

Given the differential function of the dorsal and ventral hippocampus in learning and memory processes, it can be expected that profound differences also occur in long-term potentiation (LTP), a key cellular process of learning and memory^7–9^. Indeed, several studies reported dorsoventral differences for the CA1 region in rats and mice, where LTP was found to have a significantly larger amplitude in dorsal than in ventral segments, both *in vivo* ^10^ and *in vitro* ^11–15^. Less is known about whether a similar dorsal-ventral difference is present in the DG. One *in vivo* study in rats described a higher propensity to express LTP in the intermediate than dorsal DG^16^, but the ventral DG was not evaluated.

Here, we employed multi-electrode array (MEA) recordings of short- and long-term synaptic plasticity in the DG of young and middle-aged mice, to examine whether this hippocampal subfield shows a clear dorsoventral functional segregation and whether it is subject to age-dependent changes. In addition, we assessed potential differences in inhibitory tone between hippocampal segments by pharmacological GABA_A_-receptor antagonism and measurements of miniature inhibitory postsynaptic currents (mIPSCs) and tonic inhibition.

## Results

### Distinct electrophysiological properties in dorsal, intermediate and ventral dentate gyrus

**Figure 1:**
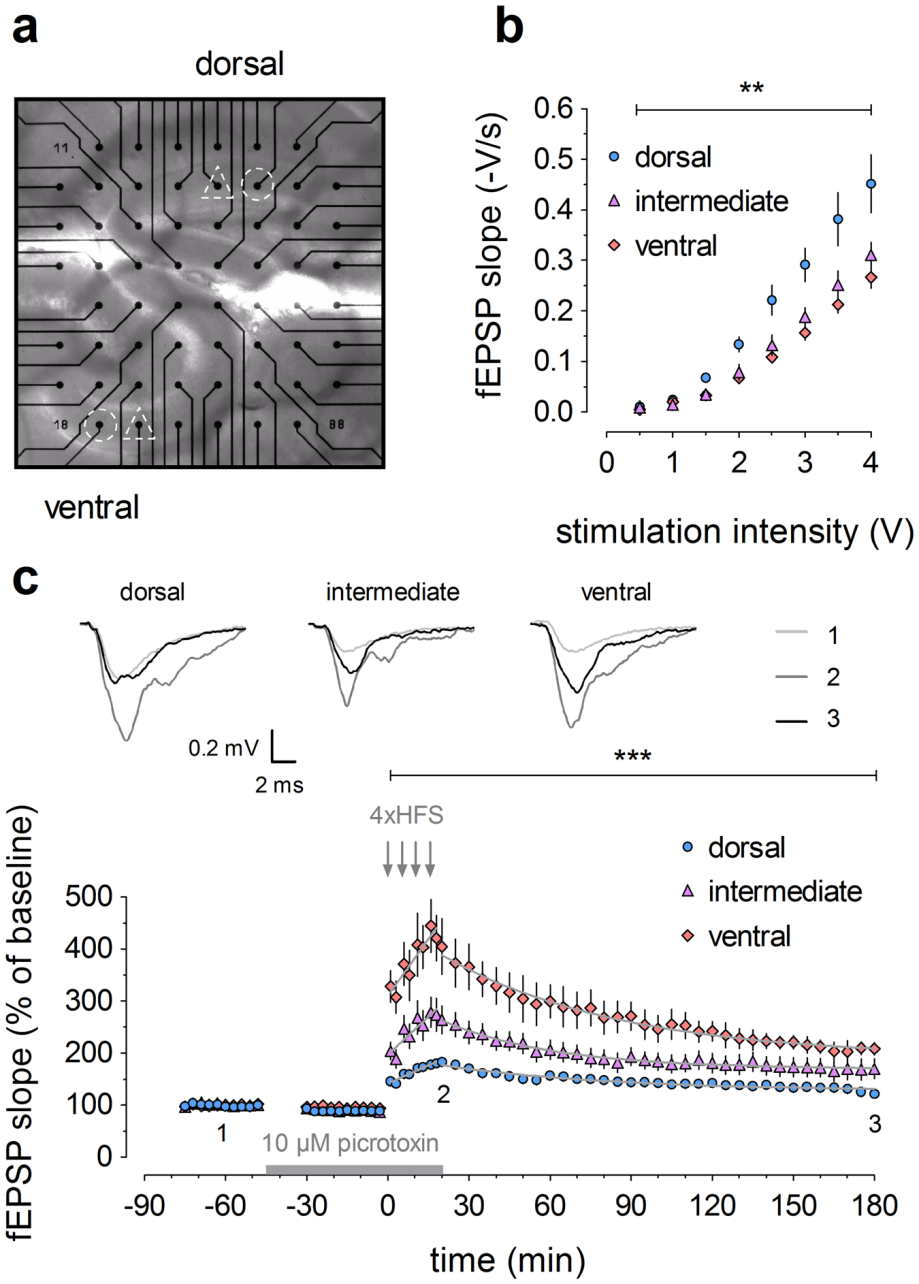
Young mice (2–3 months old) exhibit distinct electrophysiological properties in dorsal, intermediate and ventral dentate gyrus. (**a**) Image of a dorsal (top) and ventral (bottom) hippocampal slice positioned simultaneously on the multi-electrode array (MEA), zoomed in on the dentate gyrus. Selected stimulation electrodes (triangles) target the medial perforant path. Recording channels of interest (circles) are also indicated. (**b**) Input/output relationships of dorsal (n = 9), intermediate (n = 6) and ventral (n = 6) hippocampal slices indicate that basal synaptic transmission gradually decreases along the dorsoventral axis. (**c**) High-frequency stimulation (HFS; four trains of 1 s duration at 100 Hz) in dorsal (n = 9), intermediate (n = 8) and ventral (n = 6) slices induces LTP with a higher magnitude and distinct induction and decay kinetics in the ventral compared to intermediate and dorsal DG. Inset shows representative traces of field excitatory postsynaptic potential (fEPSP) for baseline, 20 min post-HFS and 180 min post-HFS, as indicated by numbers 1–3. 10 μM picrotoxin was added to the ACSF after baseline and until the end of HFS, as indicated by the bar. The grey lines superimposed on the data points denote the best-fitted functions for the induction of potentiation and subsequent decay. See Supplementary Table S1 for all equation parameters. Two-way RM-ANOVA was used for statistical analysis (** indicates *p* < 0.01 and *** indicates *p* < 0.001).

In a first series of experiments, we prepared acute dorsal (D), intermediate (I) and ventral (V) hippocampal slices from young (2–3 months old) C57BL/6J mice (Fig. 1). Slices were kept in an incubation chamber for at least 90 min and then placed on standard planar multi-electrode array (MEA) chips containing 60 electrodes (Multi Channel Systems, Germany). One or two slices were placed on the MEA (Fig. 1a), and for each slice, an electrode targeting the medial perforant path in the DG was selected. Field excitatory postsynaptic potentials (fEPSPs) were evoked by biphasic, constant voltage test pulses. Analysis of input/output curves (Fig. 1b) revealed not only the expected significant effect of stimulus intensity (*F*_7,126_ = 144.60, *p* < 0.0001), but also significant differences in basal synaptic transmission between the hippocampal regions (main effect of region: *F*_2,18_ = 8.21, *p* = 0.0029; interaction between region and intensity: *F*_14,126_ = 5.58, *p* < 0.0001). As depicted in Fig. 1b, basal synaptic transmission in dorsal slices was significantly higher compared to slices from the intermediate or ventral hippocampus (post hoc tests, for D vs I: *p* < 0.01; D vs V: *p* < 0.001; I vs V: ns).

Next, we investigated long-term potentiation (LTP) induced by high-frequency stimulation (HFS; 100 Hz for 1 s, repeated four times with 5 min interval) and with 10 μM picrotoxin (PTX) added to the recording solution (Fig. 1c). Strikingly, the initial magnitude of potentiation was much larger in ventral than in dorsal or intermediate slices (at 20 min, V: 404.66 ± 53.45%, D: 182.71 ± 4.33%, I: 263.53 ± 27.06%; one-way ANOVA: *F*_2,20_ = 13.87, *p* = 0.0002), resulting in a significant overall effect of hippocampal segment (0–180 min, two-way RM-ANOVA: *F*_2,20_ = 14.73, *p* = 0.0001). The LTP time-course in intermediate DG fell right in between and was significantly different from its dorsal and ventral neighbour segments (post hoc tests for D vs V, D vs I and I vs V: *p* < 0.001). However, as illustrated in Fig. 1c, the differences between segments were most pronounced during LTP induction and diminished thereafter. To investigate in detail whether potentiation parameters other than magnitude also varied across longitudinal segments, we analysed the induction phase by linear regression and found significant differences between segments in the y-intercept (extra sum-of-squares F test: *F*_2,178_ = 23.71, *p* < 0.0001), but not in the rising slope (*F*_2,178_ = 2.55, *p* = 0.0812) (for all equation parameters, see Supplementary Table S1). When only the two most extreme LTP curves were compared, i.e. the ones of the dorsal and ventral segment, the analysis revealed significantly different slopes (*F*_1,116_ = 5.37, *p* = 0.0222) and confirmed the difference in intercept described above (*F*_1,116_ = 50.59, *p* < 0.0001). Next, LTP decay kinetics were evaluated by nonlinear regression and fitted with a one-phase exponential decay function, as described before by us and others^17, 18^. This analysis confirmed significantly different plateau values (*F*_2,750_ = 4.77, *p* = 0.0087), which is a consequence of the different magnitude of LTP, but unveiled similar decay time constants τ (tau) (*F*_2,750_ = 0.66, *p* = 0.5185) (Supplementary Table S1).

### Dorsoventral differences in electrophysiological parameters are age-dependent

**Figure 2:**
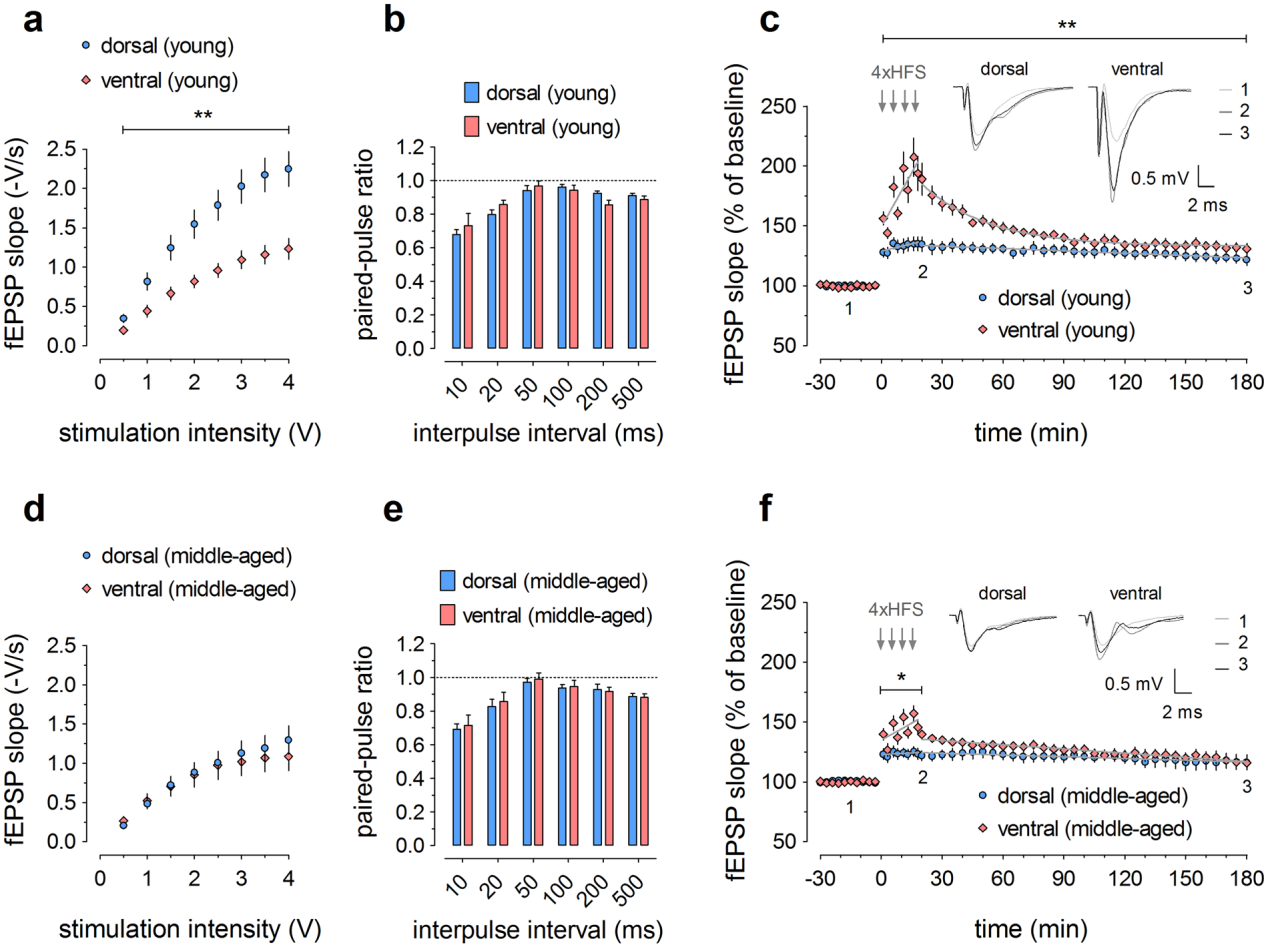
Distinct electrophysiological properties in dorsal and ventral dentate gyrus of young (**a–c**) and middle-aged (**d–f**) mice. (**a**,**d**) Input/output curves show a dorsoventral gradient in young (sample sizes: dorsal = 11, ventral = 10) but not in middle-aged mice (dorsal = 9, ventral = 7). (**b**,**e**) Paired-pulse ratios are similar between dorsal and ventral DG, both in young (dorsal = 11, ventral = 9) and middle-aged mice (dorsal = 9, ventral = 7). (**c**) Long-term potentiation in young mice (dorsal = 7, ventral = 9) is characterized by dorsoventral variation in both the induction and maintenance phase. (**f**) In middle-aged mice (dorsal = 8, ventral = 6), only the induction phase shows dorsoventral differences, whereafter the curves of dorsal and ventral LTP rapidly converge. Representative signal traces are shown for baseline, 20 min post-HFS and 180 min post-HFS, as indicated by numbers 1–3. In this series of recordings, no picrotoxin was used. The grey lines superimposed on the data points represent the best-fitted functions for the induction and decay phases. See Supplementary Tables S2 and S3 for all equation parameters. RM-ANOVA was used for statistical analysis (* indicates *p* < 0.05 and ** indicates *p* < 0.01).

In a second series of experiments, we employed the recently developed ‘multi-electrode-optrode array’ (MEOA) from imec (Heverlee, Belgium)^19^, which features a densely-spaced, combined electrical and optical LED array and improved signal-to-noise ratio. For this study, we only utilized the electrical array. We again investigated input/output relationships and LTP from dorsal and ventral hippocampal slices, prepared as described before, but now from young (2–3 months) and middle-aged (9–12 months) mice (Fig. 2). In these experiments, we did not add any PTX to maintain a more physiological excitation/inhibition balance. We also included paired-pulse stimulation to assess a short-term, presynaptic form of plasticity.

In slices prepared from young animals, we could replicate our previous finding of higher basal synaptic transmission in the dorsal DG (*F*_1,19_ = 14.46, *p* = 0.0012; Fig. 2a). Paired-pulse ratios indicated no significant differences in short-term plasticity (Fig. 2b). Subsequent LTP experiments (Fig. 2c) showed the expected smaller LTP magnitude in the absence of PTX as compared to the experiments with 10 μM PTX before. Importantly, however, the dorsoventral difference in potentiation was maintained (*F*_1,14_ = 11.88, *p* = 0.0039). Linear regression for the induction phase (Supplementary Table S2) revealed significant differences for both the intercept (*F*_1,124_ = 5.07, *p* = 0.0261) and rising slope (*F*_1,124_ = 10.07, *p* = 0.0019), which is already apparent in Fig. 2c. These results are indicative of marked differences in potentiation induction kinetics between dorsal and ventral DG. Nonlinear regression analysis for the LTP decay phase confirmed the principal differences between both segments by yielding the best fit of ventral LTP with a one-phase exponential decay function (*F*_1,294_ = 71.97, *p* < 0.0001), as before, but dorsal LTP decay with a straight line (linear regression) (Supplementary Table S2).

When the same electrophysiological parameters were examined in middle-aged animals, we could no longer obtain any differences in basal transmission (Fig. 2d). Noteworthy, this seems to be due to an age-dependent decrease of basal synaptic effectiveness in the dorsal DG, as evidenced by a statistical comparison of dorsal input/output curves from young versus middle-aged mice (*F*_1,18_ = 10.05, *p* = 0.0053). While paired-pulse values showed the same pattern as in young mice (Fig. 2e), the dorsoventral difference in LTP magnitude was clearly reduced, and only significant during induction (0–20 min: *F*_1,12_ = 9.20, *p* = 0.0104); no longer in the maintenance phase (20–180 min: *F*_1,12_ = 0.8261, *p* = 0.3813; Fig. 2f). Linear regression analysis of the induction phase indicated that the intercepts were significantly different (*F*_1,108_ = 6.14, *p* = 0.0148), similarly as in young mice, but the slopes were the same (*F*_1,108_ = 3.65, *p* = 0.0588) (Supplementary Table S3). For both dorsal and ventral DG, the LTP decay phase was now best fitted by linear regression, with significantly different slopes (*F*_1,458_ = 7.98, *p* = 0.0049) and intercepts (*F*_1,458_ = 22.34, *p* < 0.0001) between the segments (Supplementary Table S3).

### GABA_A_-receptor antagonism differentially affects dorsal and ventral dentate gyrus

**Figure 3:**
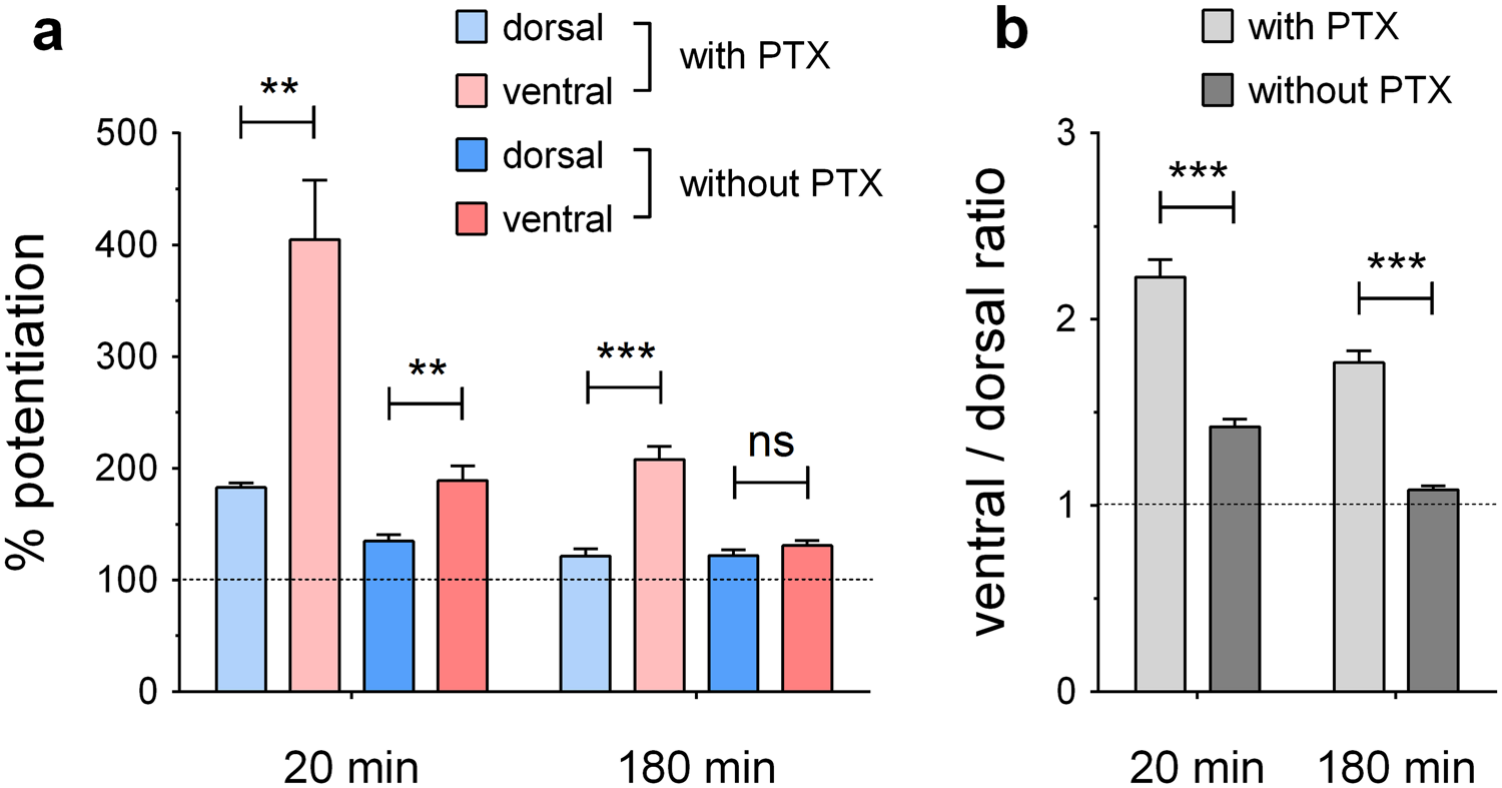
Picrotoxin (PTX) has a stronger potentiating effect on the ventral dentate gyrus in young mice (2–3 months old). (**a**) Levels of potentiation obtained at 20 and 180 min after the start of HFS, for dorsal and ventral slices, with or without the addition of PTX to the recording solution during induction (as can be derived from Figs 1c and 2c, respectively). (**b**) Comparison of mean ventral/dorsal ratios, calculated based on all possible ratios, for both conditions at 20 and 180 min. Unpaired *t* tests with Welch’s correction were used for statistical comparisons (** indicates *p* < 0.01 and *** indicates *p* < 0.001).

Next, we wanted to investigate in more detail how disinhibition by the GABA_A_-receptor antagonist picrotoxin (PTX) contributes to the observed dorsal-ventral differences in young mice. Hereto, we compared the levels of potentiation obtained at 20 min (i.e. initial potentiation, 5 min after the last HFS) and at 180 min for both conditions, with and without PTX in the recording solution (as derived from Figs 1c and 2c, respectively; Fig. 3a). We found that the addition of PTX leads to higher potentiation levels at 20 min in both regions (condition effect: *F*_1,27_ = 34.85, *p* < 0.0001; two-way ANOVA), but also that it has a markedly stronger effect on the ventral DG (interaction effect: *F*_1,27_ = 14.09, *p* = 0.0008). At 180 min, the ventral – but not dorsal – potentiation level is still greatly affected by PTX application during induction (condition effect: *F*_1,27_ = 30.76, *p* < 0.0001; interaction effect: *F*_1,27_ = 31.38, *p* < 0.0001). For simplicity, the graph in Fig. 3a only shows the region effects (left to right: *t*_5.07_ = 4.14, *p* = 0.0088; *t*_11.02_ = 3.71, *p* = 0.0034; *t*_7.93_ = 6.41, *p* = 0.0002; *t*_13.17_ = 1.33, *p* = 0.21; unpaired *t* tests with Welch’s correction). Furthermore, to directly compare potentiation levels between the two experimental conditions, we calculated mean ventral/dorsal ratios (see Methods for details). Statistical comparison revealed that this ratio was significantly affected by PTX application, both at 20 min (*t*_74.10_ = 7.88, *p* < 0.0001) and at 180 min (*t*_63.61_ = 10.61, *p* < 0.0001). These results were confirmed by an independent statistical approach using a bootstrapped resampling method (see Methods and Supplementary Fig. S1 for details). Altogether, these findings suggest a stronger sensitivity to PTX of ventral DG-LTP, likely caused by a higher GABA_A_ receptor-mediated inhibitory tone in this region compared to the dorsal DG.

### Higher frequency, but similar amplitude of miniature inhibitory postsynaptic currents in ventral compared to dorsal granule cells

**Figure 4:**
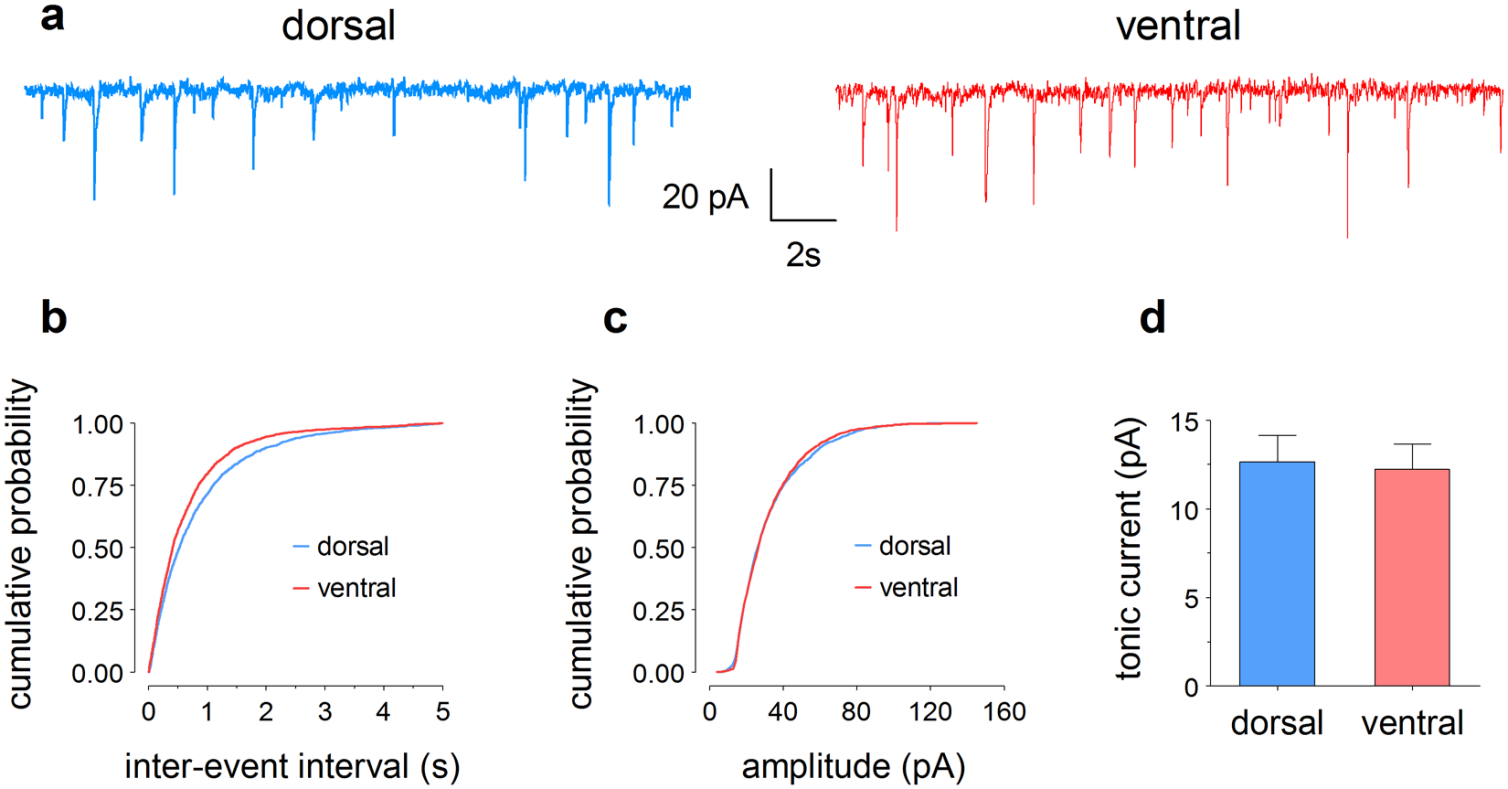
Whole-cell patch-clamp recordings reveal differences in inhibitory properties between dorsal and ventral DG granule cells. (**a**) Representative traces of miniature inhibitory postsynaptic currents (mIPSCs) of granule cells in slices from dorsal (blue) and ventral (red) hippocampus. (**b**) Cumulative probability curves indicate significantly shorter median inter-event intervals, i.e. a higher frequency of mIPSCs, in the ventral (409 ms) compared to dorsal DG (524 ms). (**c**) In contrast, the median mIPSC amplitudes are not different (dorsal: 26.6 pA, ventral: 26.9 pA). (**d**) Tonic currents in dorsal and ventral granule cells, calculated as the difference between the holding current in the absence and presence of 50 mM bicuculline, are almost identical (dorsal: 12.6 ± 1.5 pA, ventral: 12.2 ± 1.4 pA).

To further investigate the apparently higher inhibitory tone in the ventral DG, we performed measurements of miniature inhibitory postsynaptic currents (mIPSCs) in dorsal and ventral granule cells of young mice (Fig. 4a). We found that ventral granule cells have a significantly higher mIPSC frequency (shorter inter-event intervals), with a median inter-event interval of 409 ms compared to 524 ms in dorsal cells (Kolmogorov-Smirnov (KS) test, *p* < 0.0001, 17 neurons per region from 6 mice; Fig. 4b). In contrast, the median mIPSC amplitudes were not significantly different (dorsal: 26.6 pA, ventral: 26.9 pA; KS test, *p* = 0.1357; Fig. 4c).

Finally, we assessed tonic inhibition by applying the GABA_A_-receptor antagonist bicuculline methbromide and measuring the shift of the baseline current. There was no difference in mean values of tonic current between dorsal (12.6 ± 1.5 pA) and ventral (12.2 ± 1.4 pA) granule cells (unpaired *t* test, *p* = 0.8392, 14 neurons per region from 6 mice; Fig. 4d).

## Discussion

In this study, we report an age-dependent dorsoventral gradient in basal synaptic transmission and LTP in the mouse DG. In young (2–3 months old) mice, we obtained markedly stronger LTP in the ventral as compared to the dorsal hippocampus. Strikingly, this difference was found to be almost faded away in middle-aged (9–12 months old) animals, apparently due to a decline in strength of ventral LTP to a similar magnitude as dorsal LTP. The larger ventral DG-LTP is diametrically opposed to studies in the CA1 region, where the dorsal segment has been reported to have a higher ability to undergo LTP^10–14^. In accordance with a dorsoventral gradient rather than a clear-cut segregation, our LTP data of the intermediate segment fall right in between those of dorsal and ventral slices, supporting the presence of an intermediate region that expresses “partly overlapping characteristics with its neighbour regions”, as proposed by Fanselow and Dong (2010)^1^. *In vivo* findings of Kenney and Manahan-Vaughan (2013) indicate the existence of a similar dorsoventral LTP gradient in the DG of rats, as they reported a lower LTP induction threshold and stronger potentiation in the intermediate compared to the dorsal part^16^.

In addition to the dorsoventral difference in LTP magnitude, we found that the LTP induction and decay kinetics vary along the dorsoventral hippocampal axis, as assessed by linear and non-linear regression analyses. Although potentiation in both regions converged into similar levels of stable potentiation from about two hours onwards, the marked differences in dynamics point to a clear dorsoventral segregation of the mechanisms that sustain LTP.

In agreement with the latter, molecular, anatomical and functional studies have reported strong differences between dorsal and ventral hippocampus (as reviewed in refs 1 and 2). For example, gene expression studies revealed dorsoventral variation for all subfields of the hippocampus^20–22^. The expression of several proteins related to synaptic transmission and signal transduction was found to differ along the dorsoventral axis^21^, providing one putative substrate for the observed LTP differences. Furthermore, the subunits of AMPA and NMDA glutamate receptors, important postsynaptic components in synaptic plasticity, are differentially distributed along the dorsoventral axis in the rat hippocampus^23^. Several other channels that are important for intrinsic excitability are also differentially expressed or activated along the dorsoventral axis^14, 24–27^. This results in decisive differences in neuronal parameters which control the excitability and firing properties, and hence the basal input/output properties and LTP magnitude obtained in response to particular induction protocols^13, 25, 28^. Although almost all of these investigations have been performed in CA1 pyramidal cells, it is reasonable to assume that similar factors underlie the different LTP properties between dorsal and ventral DG. The opposite dorsoventral gradients of LTP properties in the CA1 region versus DG are most likely due to a different excitation/inhibition balance.

In line with this, the DG is known to maintain a higher inhibitory tone than other hippocampal subfields^29, 30^. Noteworthy, we obtained a higher LTP magnitude in the ventral DG despite the fact that most types of GABAergic interneurons were reported to increase in number along the dorsoventral axis^31, 32^. This underlines that the magnitude of potentiation does not depend solely on the sheer number of interneurons but rather on the overall excitatory/inhibitory balance. While detailed investigations of dorsovental differences regarding inhibitory processes in the DG are missing, studies in the CA1 region suggest marked differences in GABA_A_-receptor mediated recurrent inhibition^33^, which are best explained by dorsoventral differential expression of particular subunits, leading to contrasting properties of the synaptic pentameric GABA_A_-receptor protein complexes^34^. On the other hand, distinct proportions of certain subunits (such as α4, β3 and δ) in extrasynaptic GABA_A_-receptors could result in dorsoventral differences in tonic inhibition^34, 35^, but our measurements did not provide evidence for this.

We did find a higher frequency of mIPSCs in ventral granule cells, which points to a higher presynaptic GABA release in this segment^36^, and is in agreement with the stronger potentiating effect of PTX application on ventral DG-LTP. Noteworthy, the low concentration of PTX (10 μM) used in our experiments did not affect baseline responses and caused only partial inhibition.

In the present study, we did not detect any differences in paired-pulse responses between dorsal and ventral DG. However, substantial differences in paired-pulse inhibition and facilitation have been reported for rats *in vitro* ^37^ and *in vivo* ^16^.

Another factor that may contribute to the dorsoventral LTP gradient in DG and its opposite direction in CA1 is adult neurogenesis, since the DG is one of the rare neurogenic niches in the adult brain. A pool of radial glia-like stem cells in the subgranular zone regularly undergoes asymmetric divisions and gives rise to new neurons, even in aged animals. It is hypothesized that these adult-born cells play an important role in hippocampal functioning and synaptic plasticity, both during maturation and after integration into pre-existing networks (as reviewed in ref. 38). The maturation itself takes several weeks, during which the young neurons display an enhanced ability to undergo LTP^39, 40^. Interestingly, the induction of LTP without the use of GABA-receptor antagonists (and thus under more physiological conditions) was reported to depend on young, adult-born neurons, in contrast to LTP induced in the presence of PTX^41, 42^. As such, the reduced ventral DG-LTP that we reported here in middle-aged animals, recorded in the absence of PTX, may directly reflect decreased neurogenesis in this segment. Indeed, the dynamics of neurogenic processes are reported to differ between the dorsal and ventral hippocampus. The maturation of new granule cells in the ventral DG was shown to be slower^43, 44^, giving them a wider time window to express enhanced plasticity. Furthermore, although the net neurogenic capacity is similar between both regions, adult neurogenesis ﻿happens﻿ more continuously and less disciplined in the dorsal hippocampus^45^. Dorsal adult-born cells were shown to be crucial for contextual discrimination, whereas those in the ventral DG are required for anxiolytic effects^46, 47^. Interestingly, manipulations of adult neurogenesis, e.g. by antidepressants, environmental enrichment or physical exercise, may selectively target either the dorsal or ventral segment^43, 47, 48^. All of these aspects seem to reflect a different functional role for adult neurogenesis in the dorsal and ventral hippocampal segments, which may contribute to the observed differences in synaptic plasticity.

Since an age-dependency of hippocampal dorsoventral segmentation has not been reported before, the underlying mechanisms for the marked gradient in LTP seen in young but not in middle-aged mice are difficult to delineate. Many studies agree that electrophysiological properties of neurons such as resting membrane potential, membrane time constant and input resistance remain the same during normal ageing, but some report an increase in Ca^2+^ conductance and after-hyperpolarizing potential in aged neurons that may cause disturbances in Ca^2+^ homeostasis and reduced neuronal excitability (as reviewed in refs 49 and 50). With regard to the effects of ageing on synaptic plasticity, published data support the view that the induction of LTP in CA1, CA3 and DG is unchanged in response to strong high-frequency standard protocols, but that LTP maintenance tends to be impaired. When weak induction protocols are used, aged animals often show a deficit in LTP induction^50^. In previous studies, we found impaired LTP maintenance in the CA1 region of 20 months old mice *in vitro* ^51^, but no LTP deficits in the dorsal DG of middle-aged 12–16 months old rats *in vivo* ^52^, in line with the findings reported here. Recent work of our laboratory indicated that ageing goes along with certain changes in the lipid composition of neuronal membranes that lead to an attenuation or impairment of LTP and other types of synaptic plasticity^51, 53^, and are associated with cognitive deficits, but whether these age-dependent changes in membrane composition differ between dorsal and ventral hippocampal segments is unknown. Hence, which mechanisms underlie the age-dependent diminution of the dorsoventral LTP gradient remains elusive.

The human hippocampus can also be segmented into an anterior (ventral) and posterior (dorsal) part, and longitudinal differences in anatomy and function are very likely conserved between rodents and humans^54, 55^. Many neuroimaging studies have reported structural and functional connectivity differences along the longitudinal human hippocampal axis (as reviewed in refs 2 and 54). Considering that DG-LTP could also be induced in human hippocampal tissue, where it was shown to have nearly identical properties as in rodents^56, 57^, it is tempting to speculate that a similar dorsoventral gradient in DG-LTP is present in humans.

Ageing also has a profound impact on the dorsoventral functional specialization in humans and it appears to predominantly target the DG over other hippocampal subfields^58, 59^. Moreover, the anterior (ventral) segment seems to undergo the largest age-related volume loss^60, 61^. In line with this, functional connectivity was shown to undergo a shift from anterior to posterior hippocampal dominance during ageing^62^.

A human functional anterior-posterior gradient also has major clinical implications, given the broad variety of hippocampus-related neurological disorders that may preferentially target either segment. For example, cognitive decline, dementia and Alzheimer’s disease are all associated with a profoundly higher atrophy in the anterior segment^61, 63–65^. This is not surprising given that ageing also preferentially affects this segment, as discussed above, and these conditions are typically age-dependent. Other diseases that can be linked to a specific part of the hippocampal longitudinal axis include depression, medial temporal lobe epilepsy, schizophrenia and ischemia (as reviewed in refs 2, 59 and 66). The results of our study may directly reflect a higher vulnerability of the ventral DG to age- and disease-related disturbances, possibly including or being (partially) caused by reduced adult neurogenesis. Altogether, the dorsal-ventral (posterior-anterior) distinction is an important factor to take into account when studying underlying mechanisms, diagnostics and potential treatments.

Finally, all above-mentioned disorders are thought to be based on synaptic dysfunctions that are becoming overt as deficits in synaptic plasticity^67, 68^. It will be interesting to examine in future research whether the dorsoventral differences in synaptic plasticity and their age- and inhibition-dependency observed in this study, are maintained in animal models for human diseases and in human patients.

## Methods

### Animals

C57BL/6J mice were bred and raised in our own animal facility. All animals were group-housed with *ad libitum* access to food and water, and kept at constant temperature and humidity in a normal 12 h light/dark cycle (lights on from 8 am–8 pm). In MEA experiments, young (2–3 months old) and middle-aged (9–12 months old) mice of both genders were used and data were pooled, since preliminary experiments indicated no gender-influences on the examined parameters. In patch-clamp experiments, only young (2–3 months old) male mice were used. The housing conditions and procedures to prepare acute brain slices were approved by the KU Leuven Ethical Committee (P203/2012) and in accordance with European Directive 2010/63/EU.

### Acute hippocampal slice preparation

Mice were rapidly killed by cervical dislocation followed by decapitation. The brain was immediately removed and submerged in ice-cold artificial cerebrospinal fluid (ACSF), saturated with carbogen gas (95% O_2_, 5% CO_2_). ACSF consisted of (all in mM): 124.0 NaCl, 4.9 KCl, 1.3 MgSO_4_, 2.5 CaCl_2_, 1.2 KH_2_PO_4_, 25.6 NaHCO_3_ and 10.0 glucose (pH 7.4). After isolating the hippocampus, transverse slices (300 µm thick) were prepared using a custom-made tissue chopper, alternatingly starting from the dorsal or ventral pole. Both left and right hippocampi were used, with a similar left/right ratio in all experimental groups. Slices were placed into different sectors of an incubation chamber based on their origin relative to the dorsoventral axis, constantly supplied with carbogen and allowed to recover at room temperature for at least 90 min.

### Multi-electrode array recordings

After incubation, up to two slices were placed simultaneously on a multi-electrode array (MEA) chip and perfused with ACSF (same composition as above) at 2.5 ml/min and a constant temperature of 32 °C. Slice positioning was guided by an inverted light microscope (Leica DM IL LED, Leica, Germany) or upright stereo light microscope (Olympus SZ61, Olympus, Japan) and pictures were taken for each experiment using a 3.0 MP microscope camera (Moticam 3, Motic, China). A harp slice grid (ALA Scientific Instruments, USA) was put on top of the slice(s) to assure immobilization and optimal contact with electrodes. Two types of MEAs, both containing 60 electrodes, were used: the standard planar 60MEA200/30iR-Ti type from Multi Channel Systems (Germany) and the recently developed multi-electrode-optrode array (MEOA) from imec (Belgium)^19^. Both types of MEAs contain 60 electrodes in a 8 × 8 layout, and are compatible with the MEA system by Multi Channel Systems (Germany), consisting of a MEA1060-BC amplifier, SG4002 stimulus generator, TC02 temperature controller and software (MEA_Select, MC_Stimulus, MC_Rack). Data streams were sampled at 10 kHz. For each slice, a single electrode was selected for stimulation. Biphasic, constant voltage pulses (100 µs pulse-width) were applied to evoke field excitatory postsynaptic potentials (fEPSPs) from the medial perforant path in the suprapyramidal blade of the DG. Correct placement in this pathway was confirmed by the observation of paired-pulse depression responses at an interpulse interval of 40 ms. Hereafter, an input/output curve with stimulation intensities ranging from 0.5 to 4.0 V (in steps of 0.5 V), each applied twice with 30–120 s interval, was established. The intensity evoking 40% of the maximal fEPSP slope was used for further stimulation. Where mentioned, an extended series of paired-pulse stimulation, at interpulse intervals of 10, 20, 50, 100, 200 and 500 ms, was recorded. After obtaining a stable baseline of at least 30 min, LTP was induced by four trains of high-frequency stimulation at 100 Hz, 1 s in duration and 200 µs pulse-width, with 5 min interval. In specified experiments, picrotoxin (PTX, Sigma-Aldrich; dissolved in dimethyl sulfoxide (DMSO) to working concentration of 100 mM) was added to the ACSF (final concentration: 10 μM PTX, 0.01% DMSO) right after the baseline, to reduce GABA_A_ receptor-mediated inhibition and evoke robust LTP. In this case, a second 30-min baseline was recorded to allow confirmation that PTX did not affect baseline responses. Right after LTP induction, the recording solution was switched back to regular ACSF.

### Multi-electrode array data analysis

Raw data were extracted using MC_Rack software (Multi Channel Systems, Germany) in replayer mode. Although all electrodes of the MEA served as recording channels, we focused our analyses on the single channel adjacent to the stimulation electrode (in the anterograde direction of the perforant path). To obtain fEPSP slope values, the region of interest was set from peak-to-peak and the software calculated the average slope within 10–90% of this region. Paired-pulse ratios were calculated by dividing the slope of fEPSP_2_ by the slope of fEPSP_1_. In LTP experiments, all data points were normalized to the average baseline slope.

### Patch-clamp recordings

Patch-clamp whole-cell measurements of miniature inhibitory postsynaptic currents (mIPSCs) and tonic inhibition from DG granule cells were performed using a MultiClamp 700B patch-clamp amplifier and data were collected using pClamp software (Axon Instruments, USA). Transverse slices (400 µm thick) were prepared correspondingly from the dorsal (at an angle of ~10 degrees para-sagittal) and ventral (at ~10 degrees para-horizontal) hippocampus using a vibratome (Microm HM 650 V, Thermo Scientific, USA). The glass microelectrodes for patching were filled with a solution containing (in mM): 140 CsCl, 10 Na-HEPES, 10 EGTA, 2 MgATP, 5 QX-314, pH 7.3 (pipette resistance 3–4 MΩ). Voltage-clamp recordings from DG granule cells were made at holding potential −60 mV while the ACSF was supplemented with CNQX (20 µM), AP5 (40 µM) and TTX (1 µM).

Subsequently, tonic inhibition was measured in the same DG granule cells by adding 50 µM bicuculline methbromide (BIC) to the ACSF superfusate. The value of tonic current was calculated as the difference in baseline current before and after the addition of BIC at holding potential −60 mV, as described previously^69^. During acquisition, all data were low-pass filtered at 2 kHz and sampled at 10 kHz.

### Patch-clamp data analysis

As previously described^69^, off-line amplitude and frequency analysis of mIPSCs was performed using MiniAnalysis software 6.07 (Synaptosoft, USA). Mean tonic current values were obtained from Gaussian fits to all-point amplitude histograms (not shown) and the difference between peak values of two simulated Gaussians was used as a measure of the baseline tonic current.

### Statistics

All results were plotted in graphs and analysed statistically using GraphPad Prism 5.01 (GraphPad Software, USA), unless otherwise specified. Where parametric statistical tests are used, the D’Agostino-Pearson normality test was first applied to confirm normal distribution of the data. In all tests, *p* < 0.05 was considered significant. Data are presented as mean ± standard error of the mean (SEM) and n refers to the number of animals tested.

For input/output curves, paired-pulse ratios and LTP induction, (two-way) repeated measures analysis of variance (RM-ANOVA) was used with hippocampal region as between-subjects factor, intensity/interval/time as within-subjects factors, and Bonferroni’s post hoc test for multiple comparisons.

To analyse curve fits, the extra sum-of-squares *F* test was used.

The ventral/dorsal ratios were analysed by two independent statistical methods: (1) unpaired *t* test with Welch’s correction to compare the average ratios as calculated from all possible ratios (see Fig. 3), and (2) resampling by bootstrapping, using a custom-written script in the open-source statistical programming language R (R Foundation for Statistical Computing, Austria, http://www.r-project.org) (see Supplementary Fig. S1). The resampling procedure was as follows: for both conditions (with and without PTX), we randomly sampled six pairs from ventral and dorsal measurements, which were used to obtain ventral/dorsal ratios (thereby modelling an experiment with paired design and 6 subjects). The ratio obtained from the condition without PTX was then subtracted from the ratio with PTX. This procedure was repeated 10^6^ times to generate a distribution of ratio differences (with – without PTX). In addition, we obtained maximum likelihood estimates for the parameters of a normal distribution (Supplementary Fig. S1).

To compare the cumulative probabilities of mIPSC inter-event intervals and amplitudes, statistical significance was determined by two-tailed Kolmogorov-Smirnov (KS) test using MiniAnalysis software. Tonic inhibition between dorsal and ventral slices was compared statistically by unpaired *t* test. Equal numbers of recordings from dorsal and ventral hippocampus were made from each mouse (but not more than 3 pairs). In total, for each hippocampal segment, we measured mIPSCs in 17 neurons from 6 mice, and assessed tonic inhibition in 14 neurons from 6 mice.

### Data Availability

The datasets generated and analysed in this study are available from the corresponding author on reasonable request.

## Electronic supplementary material

Supplementary Information
