# Supplementary material for: Distinct Properties of Long-Term Potentiation in the Dentate Gyrus along the Dorsoventral Axis: Influence of Age and Inhibition
Source: Sci Rep. 2017 Jul 11;7:5157. doi: 10.1038/s41598-017-05358-1 (PMC5506024; doi:10.1038/s41598-017-05358-1)
Supplement: Supplementary file 1 — Supplementary Information [file 41598_2017_5358_MOESM1_ESM.pdf]

SUPPLEMENTARY INFORMATION

Distinct Properties of Long-Term Potentiation in the Dentate Gyrus along the Dorsoventral axis: Influence of Age and Inhibition

An Schreurs, Victor Sabanov & Detlef Balschun

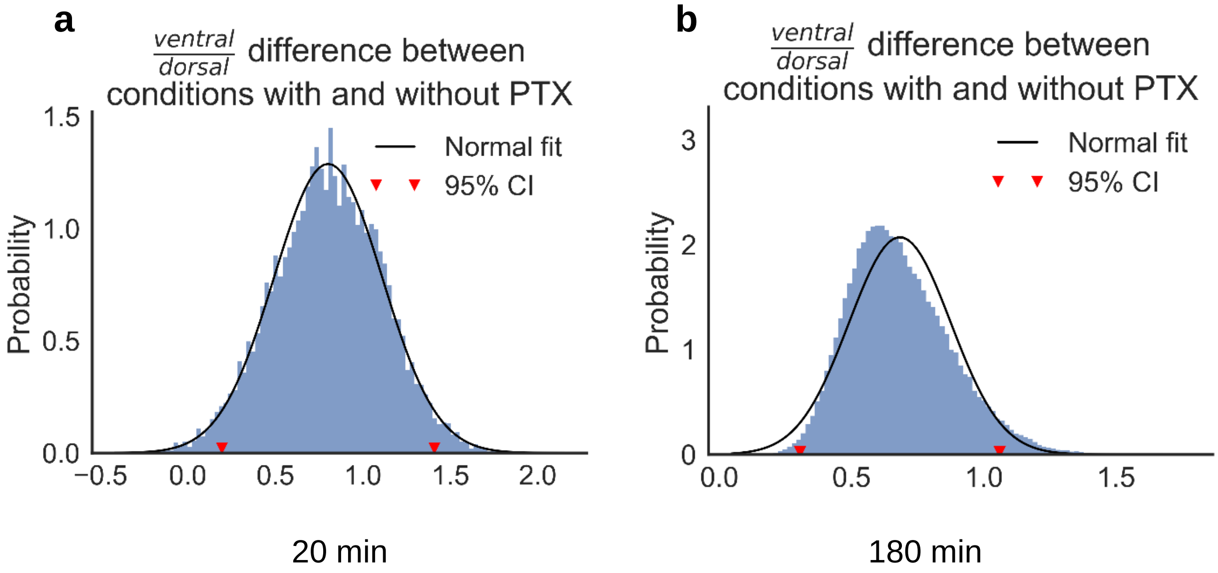

Supplementary Figure S1. Potentiation levels obtained at 20 and 180 min post-HFS (see **Fig. 3a**), were resampled by bootstrapping to compare the influence of PTX on both regions (see Methods for details). Briefly, six pairs of ventral and dorsal data points were randomly sampled from the datasets obtained with and without PTX. The difference between the average ventral/dorsal ratios thus obtained was calculated (with – without PTX), and this was repeated 10^6 times to obtain a distribution of the ratio differences.

(**a**) Distribution for the ratio differences at 20 min (mean ± standard deviation: 0.80 ± 0.30).

(**b**) Distribution for the ratio differences at 180 min (mean ± standard deviation: 0.60 ± 0.13).

Red markers indicate the 95% confidence interval (CI) of the distributions. In both cases, this interval does not contain 0, suggesting that the probability to observe a ratio difference of 0 is extremely low.

	dorsal		intermediate		ventral	
	best-fit ± SE	[95% CI]	best-fit ± SE	[95% CI]	best-fit ± SE	[95% CI]
Induction phase: straight line $y = y_{intercept} + slope*x$						
$y_{intercept}$	141.5 ± 2.57	[136.4, 146.6]	196.5 ± 17.49	[161.5, 231.5]	309.8 ± 28.84	[251.7, 367.9]
slope	2.31 ± 0.23	[1.85, 2.78]	4.87 ± 1.58	[1.71, 8.03]	7.27 ± 2.61	[2.02, 12.52]
Decay phase: one phase exponential decay $y = (y_0 - plateau)*exp(-x/τ) + plateau$						
y_0	194.8 ± 6.62	[181.8, 207.7]	320.9 ± 36.18	[250.0, 391.8]	457.9 ± 38.45	[382.5, 533.2]
plateau	127.1 ± 3.83	[119.5, 134.6]	169.3 ± 7.79	[154.0, 184.6]	191.2 ± 23.87	[144.4, 238.0]
τ (tau)	63.87 ± 16.55	[45.02, 109.9]	41.25 ± 22.44	[25.22, 113.18]	65.85 ± 36.57	[40.12, 183.48]

Supplementary Table S1. Curve fitting parameters obtained in linear and nonlinear regression analysis of the potentiation induction and decay phase, respectively, in dorsal, intermediate and ventral slices. For each parameter, the best-fit value, standard error (SE) and 95% confidence interval (CI) are listed. Visual representations of the curve fits are shown in **Fig. 1c**.

	dorsal			ventral	
	best-fit ± SE	[95% CI]		best-fit ± SE	[95% CI]
Induction phase: straight line $y = y_{intercept} + slope*x$					
$y_{intercept}$	128.75 ± 3.46	[121.82, 135.69]	$y_{intercept}$	149.18 ± 7.52	[134.16, 164.19]
slope	0.42 ± 0.31	[–0.21, 1.04]	slope	3.02 ± 0.68	[1.66, 4.38]
Decay phase:					
straight line<br>$y = y_{intercept} + slope*x$			one phase exponential decay<br>$y = (y_0 - plateau)*exp(-x/τ) + plateau$		
$y_{intercept}$	134.80 ± 1.70	[131.47, 138.14]	y_0	227.77 ± 11.73	[204.79, 250.75]
slope	–0.06 ± 0.02	[–0.09, –0.03]	plateau	132.86 ± 1.57	[129.79, 135.93]
			τ (tau)	33.23 ± 3.61	[26.16, 45.53]

Supplementary Table S2. Curve fitting parameters obtained in linear and nonlinear regression analysis of the potentiation induction and decay phase, respectively, in dorsal and ventral slices of young mice (2-3 months old). For each parameter, the best-fit value, standard error (SE) and 95% confidence interval (CI) are listed. Visual representations of the curve fits are shown in **Fig. 2c**.

	dorsal			ventral	
	best-fit ± SE	[95% CI]		best-fit ± SE	[95% CI]
Induction phase: straight line $y = y_{intercept} + slope*x$					
$y_{intercept}$	122.74 ± 2.87	[117.00, 128.48]	$y_{intercept}$	134.94 ± 4.21	[126.46, 143.43]
slope	0.10 ± 0.26	[–0.42, 0.61]	slope	0.95 ± 0.38	[0.18, 1.71]
Decay phase: straight line $y = y_{intercept} + slope*x$					
$y_{intercept}$	125.39 ± 2.00	[121.48, 129.30]	$y_{intercept}$	138.01 ± 1.56	[134.96, 141.07]
slope	–0.05 ± 0.02	[–0.08, –0.01]	slope	–0.12 ± 0.01	[–0.14, –0.09]

Supplementary Table S3. Curve fitting parameters obtained in linear and nonlinear regression analysis of the potentiation induction and decay phase, respectively, in dorsal and ventral slices of middle-aged mice (9-12 months old). For each parameter, the best-fit value, standard error (SE) and 95% confidence interval (CI) are listed. Visual representations of the curve fits are shown in **Fig. 2f**.
